# Acute Liver Injury Following Herbal Drink Consumption With Steatosis and Immune Activation: A Case Report

**DOI:** 10.7759/cureus.79839

**Published:** 2025-02-28

**Authors:** Helai Hussaini, Aiman Waheed, Olaniyi Fadeyi, Antoine Boustany, Yusuke Hashimoto, Andrew T Flint

**Affiliations:** 1 Internal Medicine, West Anaheim Medical Center (WAMC), Orange, USA; 2 Medicine, Holy Family Hospital, Rawalpindi, PAK; 3 Gastroenterology, West Anaheim Medical Center (WAMC), Orange, USA; 4 Gastroenterology, University of Florida College of Medicine – Jacksonville, Jacksonville, USA; 5 General Medicine, University of Florida College of Medicine – Jacksonville, Jacksonville, USA

**Keywords:** acute liver injury, hepatic steatosis, hepatotoxicity, herbal-induced liver injury, immune activation

## Abstract

Herbal medicines are widely used for their perceived health benefits; however, their potential for liver toxicity is well-documented. We report a case of acute liver injury (ALI) in a 32-year-old male patient following the consumption of a multi-herbal drink. The patient, with a history of cholecystectomy for gallstones and nephrolithiasis, presented with severe epigastric pain radiating to the right shoulder, nausea, high blood pressure (170/113 mmHg), and a rapid heart rate. Laboratory tests revealed significant liver damage, with aspartate aminotransferase (AST) at 1,043 U/L, alanine aminotransferase (ALT) at 1,645 U/L, and total bilirubin at 4.8 mg/dL. Ultrasound imaging showed fatty liver disease without bile duct obstruction. Autoimmune testing was negative for antinuclear antibodies (ANA) and antimitochondrial antibodies (AMA), while elevated immunoglobulin G (IgG) levels suggested immune activation as a potential mechanism. The patient improved with supportive care. This case highlights the potential liver toxicity of polyherbal remedies and underscores the importance of early recognition and management to prevent long-term complications.

## Introduction

Acute liver injury (ALI) is a significant global health concern, contributing to over two million deaths annually, with one million deaths resulting from complications such as cirrhosis and liver cancer [1]. Among its various causes, herbal and dietary supplements have increasingly been recognized as contributors to drug-induced liver injury (DILI). Herbal-induced liver injury (HILI) accounts for 16% to 20% of DILI cases in Western countries [2]. The widespread use of herbal products, particularly in regions where alternative medicine is common, poses potential risks due to the presence of multiple ingredients, lack of regulatory oversight, and possible contamination or mislabeling [3,4].

The mechanisms of HILI are complex and include direct hepatotoxicity, immune-mediated responses, and idiosyncratic reactions, making diagnosis challenging. Some cases exhibit immune activation, characterized by elevated immunoglobulin G (IgG) levels and liver biopsy findings suggestive of immune involvement [5,6].

The distinction between immune-mediated HILI and immune-mediated drug-induced liver injury (IMDILI) remains unclear, as both can present with similar biochemical and histological features. Additionally, while herbal products are not traditionally classified as pharmaceuticals, their biological effects warrant careful evaluation. The clinical spectrum of HILI ranges from asymptomatic elevations in liver enzymes to fulminant hepatic failure [6]. Diagnosis is primarily one of exclusion, requiring thorough assessment to rule out autoimmune hepatitis, viral infections, and metabolic liver diseases. The overlap in immunological markers, such as elevated IgG levels, can further complicate differentiation from autoimmune hepatitis [7]. Given these diagnostic challenges, clinicians must maintain a high level of suspicion when evaluating patients with unexplained liver injury.

Greater awareness, regulatory oversight, and systematic research are essential to identifying and mitigating the risks associated with herbal supplements to prevent severe liver injury [8].

## Case presentation

A 32-year-old male patient presented to the emergency department with severe epigastric pain radiating to the right shoulder and right upper quadrant, accompanied by nausea. The pain was similar to what he had experienced before undergoing laparoscopic cholecystectomy five years ago for acute cholecystitis. Despite the prior surgery, he reported occasional episodes of similar discomfort, which had never been as severe as the current episode. The intensity of the pain impaired his breathing, prompting medical evaluation and the administration of morphine for pain relief. His medical history also included pyelonephritis, treated with antibiotics two years ago, and nephrolithiasis diagnosed around the same time. Following his nephrolithiasis diagnosis, the patient consumed a herbal tonic (200 mL bottle), at a dose of 50 mL per day for approximately two years based on a relative’s recommendation. Symptoms began three months before the hospital presentation. The tonic contained 14 herbal ingredients: *Herniaria glabra*, *Taraxacum officinale*, *Urtica dioica*, *Solidago virgaurea*, *Equisetum arvense*, *Arctostaphylos uva-ursi*, *Juniperus communis*, *Betula pendula*,* Zea mays*, *Ononis spinosa*, *Orthosiphon stamineus*, *Foeniculum vulgare*, *Petroselinum crispum*, and *Levisticum officinale*. The patient had no medical history of hypertension, diabetes, ischemic heart disease, tuberculosis, asthma, hepatitis B or C, alcohol use, or substance abuse.

Upon examination, the patient exhibited icterus, skin discoloration, and generalized abdominal tenderness. He was tachycardic and hypertensive (170/113 mmHg), raising concerns for a cardiovascular event, which were ruled out following unremarkable cardiac and pulmonary evaluations. Laboratory findings demonstrated significant hepatocellular injury with elevated liver enzymes (aspartate aminotransferase (AST): 1,043 U/L and alanine aminotransferase (ALT): 1,645 U/L), hyperbilirubinemia (total bilirubin: 4.8 mg/dL, direct bilirubin: 1.4 mg/dL), and hypokalemia (potassium: 2.8 mmol/L). Table 1 summarizes the key laboratory findings of the patient’s hospitalization. Predominantly elevated ALT, AST, and bilirubin levels indicated hepatocellular injury. The patient’s clinical presentation, temporal association with prolonged herbal tonic consumption, and laboratory findings strongly suggested HILI as a subset of DILI.

**Table 1:** Serial laboratory investigations of the patient during the hospital stay WBC: white blood cells; RBC: red blood cells; AST: aspartate aminotransferase: ALT: alanine aminotransferase

Parameter	Day 1	Day 2	Day 3	Normal Range
WBC (x10³/µL)	15.25	9.84	8.07	4.0 - 11.0
RBC (x10⁶/µL)	6.34	5.43	5.25	4.5 - 5.9
Hemoglobin (g/dL)	18.6	16.4	16.0	13.5 - 17.5
Hematocrit (%)	54.7	47.5	46.5	41.0 - 50.0
Platelet count (x10³/µL)	319	308	288	150 - 450
AST (U/L)	1043	640	370	10 - 40
ALT (U/L)	1645	1040	600	7 - 56
Total bilirubin (mg/dL)	4.8	3.5	2.1	0.1 - 1.2
Direct bilirubin (mg/dL)	1.4	0.9	0.6	0.0 - 0.3
Alkaline phosphatase (U/L)	125	110	102	44 - 147
Potassium (K⁺, mmol/L)	2.8	3.4	3.8	3.5 - 5.0
Sodium (Na⁺, mmol/L)	140	138	139	135 - 145

**Figure 1:**
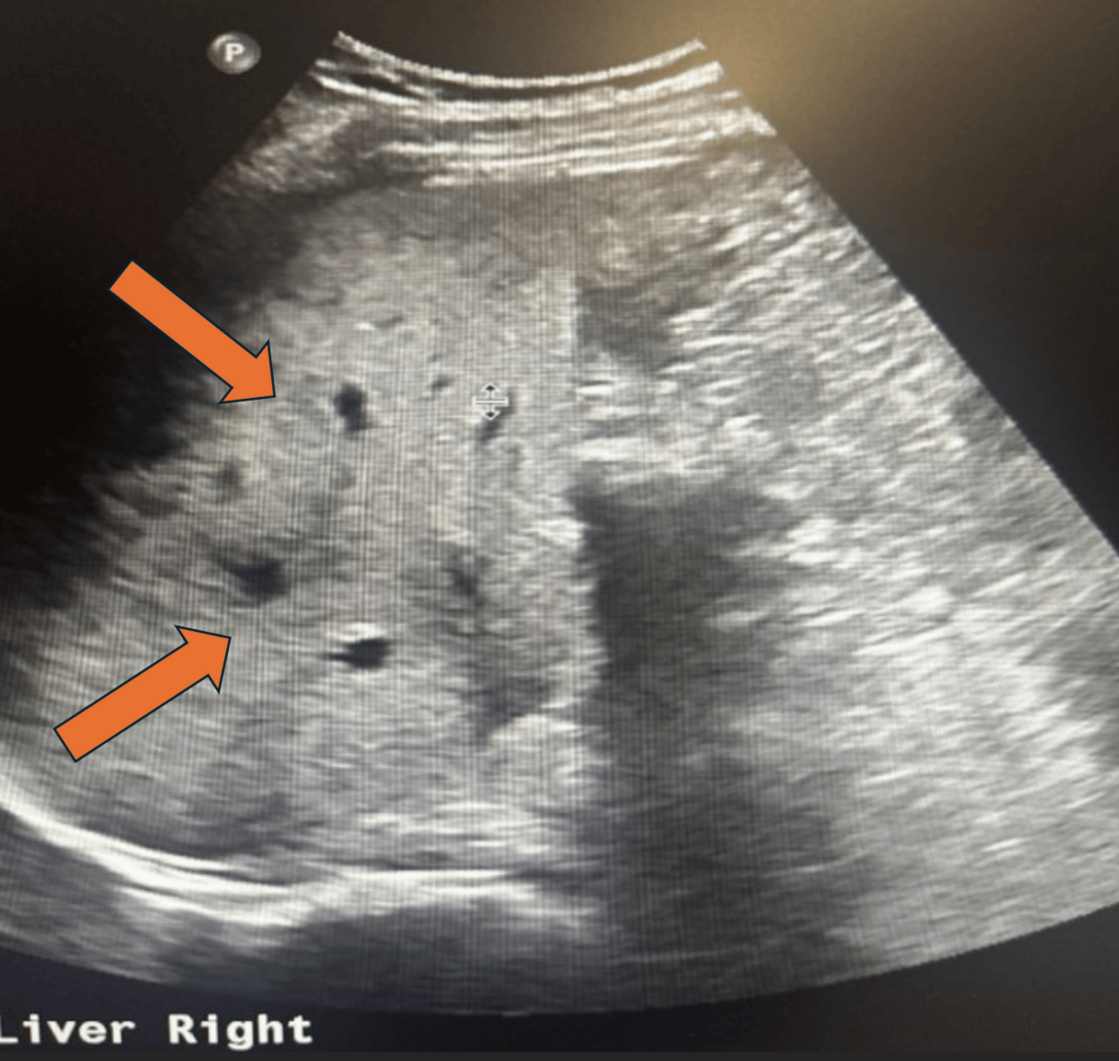
An ultrasound of the liver showing increased echogenicity, suggestive of hepatic steatosis. The orange arrows highlight enhanced liver echogenicity.

An ultrasound was performed to assess for possible biliary obstruction, given the patient's liver-associated symptoms and laboratory findings. The imaging ruled out intra- or extrahepatic biliary ductal dilatation but incidentally revealed hepatic steatosis (Figure 1).

**Figure 2:**
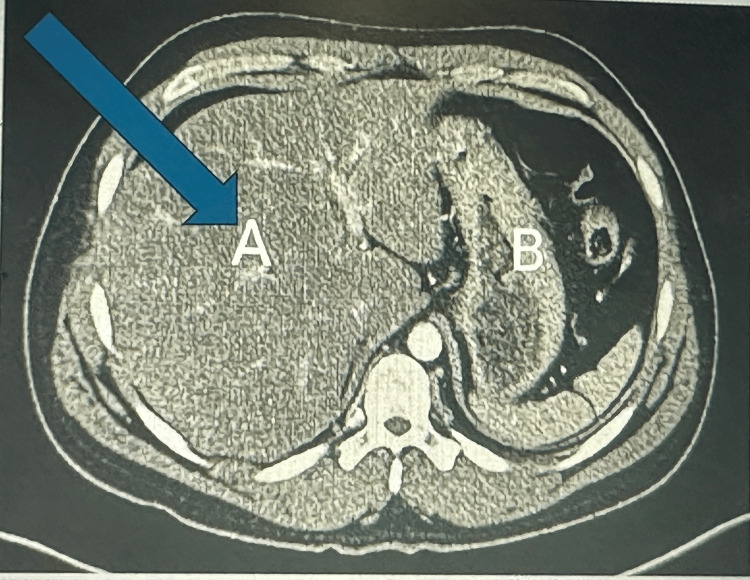
A contrast-enhanced CT scan of the liver showed diffuse low intensity, suggestive of hepatic steatosis. A: The liver shows increased echogenicity and hepatic steatosis; B: Stomach

 A contrast-enhanced CT (axial image) visualized diffuse hepatic steatosis (Figure 2).

Immunoglobulin G levels (>1600 mg/dL) were elevated, while the hepatitis panel, antinuclear antibody (ANA), smooth muscle antibody (SMA), and antimitochondrial antibody (AMA) were negative (Table 2).

**Table 2:** Serum immunological markers

Test	Result	Reference range
Antinuclear antibody (ANA)	Negative	Negative
Liver kidney microsomonal antibody	0.6 (Negative)	Positive: ≥25.0 units
Smooth muscle antibody	Negative	Negative
Antimitochondrial antibody (AMA)	Negative	Negative
Immunoglobulin G (IgG, mg/dL)	1809	700 - 1600

Supportive care was initiated, including pain management, electrolyte correction, intravenous fluid therapy, and close monitoring of liver function. The patient presented with hypokalemia (2.8 mEq/L), which was corrected with intravenous potassium chloride (KCl) over 24 hours, while other electrolytes remained within normal limits. Given the uncertainty regarding the herbal supplement’s role in electrolyte disturbances, metabolic parameters were closely monitored. The patient’s liver function tests (LFTs) initially worsened but improved over three days after discontinuing the herbal tonic, supporting a DILI etiology. The Roussel Uclaf Causality Assessment Method (RUCAM) score [9] was six points, indicating a probable diagnosis of DILI. Elevated IgG levels suggested a possible immune-mediated mechanism (IMDILI), though negative ANA and AMA tests made autoimmune hepatitis and primary biliary diseases less likely. The patient received intravenous crystalloids with potassium supplementation for 48 hours, followed by oral hydration upon stabilization. He remained hospitalized for four days, after which LFTs and electrolytes were reassessed before discharge. A one-month follow-up was advised to confirm hepatic recovery and electrolyte stability.

## Discussion

Herb-induced liver injury is a significant and growing concern due to the widespread use of herbal and alternative medications globally. This case underscores the potential hepatotoxic effects of herbal formulations, emphasizing the need for increased awareness among healthcare professionals and patients regarding their safety profiles. Immune-mediated drug-induced liver injury is an increasingly recognized subset of DILI, characterized by immune activation and hepatocyte damage. The exclusion of other etiologies such as autoimmune hepatitis, viral infections, and biliary obstruction further strengthens this conclusion. The temporal relationship between herbal drink consumption and the onset of symptoms provides strong circumstantial evidence for causality. Features of IMDILI, such as immune activation observed in this case, further support the role of immune mechanisms in liver injury.

In Asia, where herbal medicine is more prevalent, the proportion of DILI caused by herbal products is even higher. The mechanisms underlying DILI are diverse and can include direct hepatotoxicity, IMDILI, and interactions with existing liver conditions. In this case, the herbal drink’s composition of 14 ingredients could have included substances with known hepatotoxic effects, or the combination of ingredients may have had a synergistic toxic effect. Studies have identified several herbs, such as green tea extract, kava, and traditional Chinese medicines, as common culprits of DILI [10-12].

Hepatic steatosis observed in this patient’s ultrasound and CT imaging (Figures 1-2) adds another layer of complexity to the case. Hepatic steatosis, characterized by excessive fat accumulation in hepatocytes, can predispose the liver to further insults. While the patient had no history of alcohol consumption or metabolic syndrome, the herbal drink, specifically the one consumed by the patient, might have contained compounds promoting fat accumulation or disrupting lipid metabolism. Experimental studies have demonstrated that certain herbal compounds can induce oxidative stress and mitochondrial dysfunction, leading to steatosis and hepatocyte injury [13,14]. However, it is important to note that acute liver injury itself can mimic hepatic steatosis on imaging, especially in this setting. Therefore, liver histology would be required to confirm hepatic steatosis definitively, but the primary concern remains the acute hepatocellular injury, as evidenced by significantly elevated liver enzymes and hyperbilirubinemia, which align with DILI.

The immune activation observed in this case, as evidenced by elevated IgG levels, raises the possibility of an immune-mediated component to the liver injury. Although autoimmune hepatitis was ruled out based on negative ANA and AMA tests, immune-mediated mechanisms are increasingly recognized in DILI. Some herbal products can act as haptens, binding to liver proteins and triggering an immune response [15,16]. Given that the patient consumed the herbal tonic for approximately two years before developing symptoms, the chronic exposure supports the hypothesis of prolonged immune activation contributing to liver injury. The RUCAM is widely used to assess causality in DILI, but its application in HILI and IMDILI requires validation [9]. Management of DILI is primarily supportive, with discontinuation of the offending agent being the cornerstone of treatment. In this case, the patient’s symptoms and liver enzyme levels improved with symptomatic care and close monitoring. Corticosteroids may be considered in severe cases with immune-mediated features, but their use remains controversial [12]. The prognosis for DILI and IMDILI is generally favorable, with most patients recovering completely after discontinuation of the offending herbal product. However, severe cases can progress to acute liver failure, necessitating liver transplantation [17]. 

The growing popularity of herbal products necessitates stronger regulatory oversight to ensure their safety. In many countries, herbal supplements are marketed without rigorous preclinical or clinical testing, leading to potential underreporting of adverse effects. Public health campaigns should focus on educating consumers about the potential risks of herbal products and encouraging them to consult healthcare providers before use. Clinicians should remain vigilant for DILI, especially in patients with unexplained liver injury and a history of herbal supplement use. Further research is needed to identify the specific components of herbal formulations responsible for liver injury and to establish evidence-based guidelines for their safe use.

## Conclusions

Herbal drink consumption remains an under-recognized cause of ALI, as demonstrated in this case. The patient’s presentation with severe hepatocellular injury and elevated liver enzymes following the intake of a multi-ingredient herbal tonic highlights the potential for immune-mediated mechanisms, including IMDILI, or direct hepatotoxicity associated with such substances.

Although initial concerns focused on autoimmune hepatitis and biliary obstruction, further evaluation ruled out these possibilities, emphasizing the role of HILI. Prompt diagnosis, supportive care, and close monitoring are essential for managing these cases, as liver function can decline rapidly but often shows improvement with appropriate interventions. This case underscores the importance of patient history regarding alternative medicine use and raises awareness of potential adverse effects linked to herbal remedies. Clinicians should remain vigilant when evaluating patients with unexplained acute liver injuries to ensure timely diagnosis and management.
